# Bilateral Rectus Sheath Hematoma in Kidney Transplant Patient: Case Study and Literature Review

**DOI:** 10.5812/numonthly.9577

**Published:** 2013-08-14

**Authors:** Behzad Feizzadeh Kerigh, Ghodratolah Maddah

**Affiliations:** 1 Urology Department, Mashhad University of Medical Sciences, Mashhad, IR Iran

**Keywords:** Hematoma, Kidney Transplantation, Bilateral

## Abstract

****Rectus sheath hematoma usually occurs unilateral but rare cases of bilateral hematoma have been reported. Herein we report the first case of spontaneous bilateral Rectus Sheath Hematoma in the kidney transplanted patient.

## 1. Introduction

Rectus sheath hematoma (RSH) is caused due to hemorrhagia of the lower or upper epigastric arteries with or without any rupture in the rectus muscles (1). The RSH is not a common illness that is largely misdiagnosed and the most probable location is below the umbilicus (2).

Classic symptoms of the RSH appear as severe abdominal pain and a palpable mass in the senile woman usually associated with history of non-surgical trauma to the abdomen (3). Hematoma is usually unilateral, but rare cases of bilateral RSH have been reported (4-6).

The RSH resulting after kidney transplantation could be the first symptom of lymphoproliferative disease (7). As our knowledge, however, there has not been any report of the spontaneous bilateral RSH after kidney transplantation and this study intends to introduce this.

## 2. Case Study

A 49-year-old female with a decade history of the end-stage renal disease underwent the second transplant from a live donor. The patient’s first transplant was performed on the right side. After 6 years it had rejected and nephrectomy was performed. For the second transplant a J-shaped incision was performed on the left side. The left nephrectomy of the live donor was performed and the artery and vein anastomosed to common iliac artery and external iliac vein respectively. The ureter was anastomosed to the bladder in the fashion of the modified lich.

From the first day the Cyclosporine (9 mg/kg), Cellcept (2 g/d), and Prednisolone (1 mg/kg) were administrated to the patient. The patient’s creatinin was reduced to 3 mg/dL and stopped at this level. The color Doppler sonography of the transplanted kidney demonstrated RI = 0.82. The DTPA scan was performed that demonstrated decreased perfusion and poor excretion of the transplanted kidney which indicated acute rejection. The patient didn’t permit renal biopsy.

From 5th day Cyclosporine was reduced to the minimum dosage, and Cellcept was stopped. Then the thymoglobulin (1.25 mg/kg) was started, at the same time Gancyclovir for prophylaxy of *Cytomegalo virus* was initiated. In total the patient received thymoglobulin for 7 days and creatinin was reduced to 2.1 mg/dL. Upon the stoppage of the thymoglobulin the patient had fever along with productive cough. The Chest-x-Ray and HRCT were normal and after prescribing Meropenem the fever stopped.

**Figure 1.**
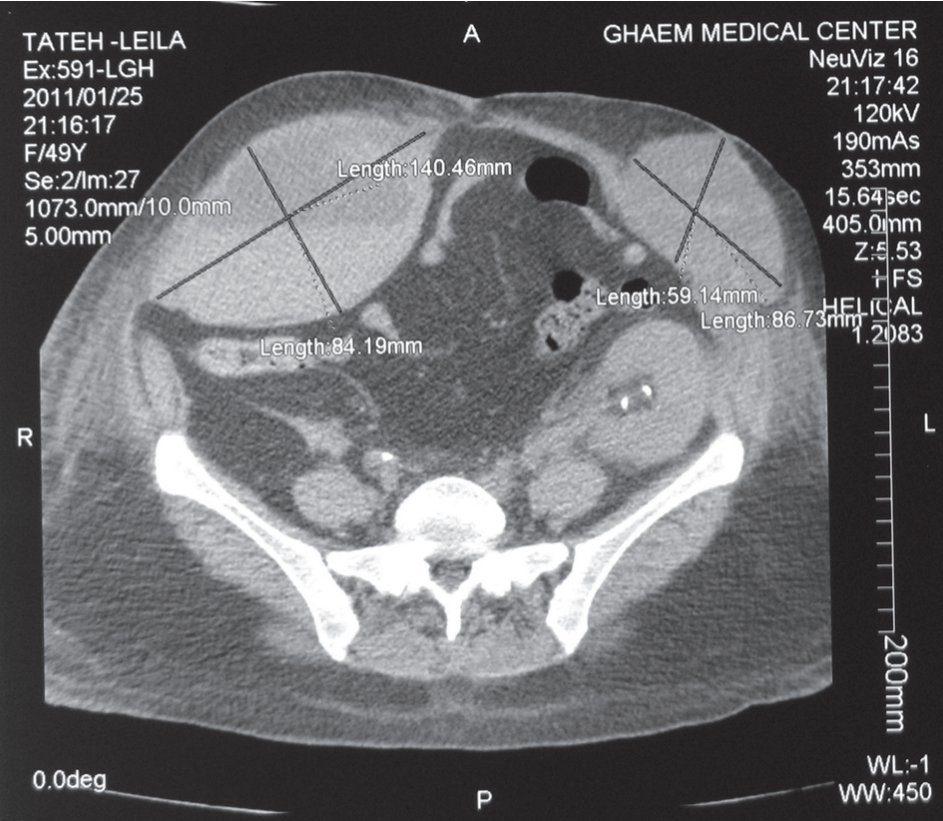
Bilateral Hematoma of Rectus Muscles Transplanted kidney apparent.

On the 25th day after the transplant the patient expressed severe pain on the right side of the abdomen (site of the first transplant) coupled with vomiting, nausea, guarding along with a palpable mass and reduction Hct and Hgb. The color Doppler sonography detected an arterial pulse in the mass. The CT scan was immediately performed which demonstrated the RSH significantly in the right side (Figure 1). The coagulation tests were normal. 

**Figure 2.**
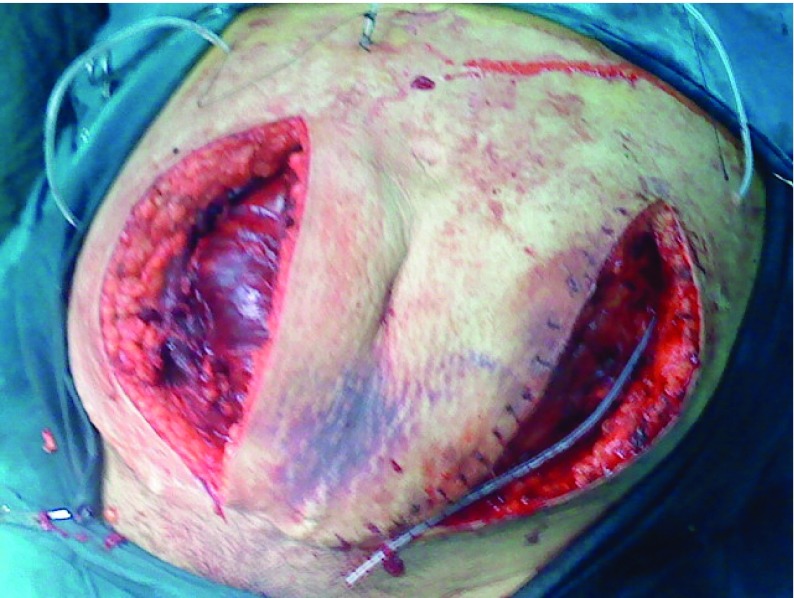
Hematoma Was Opened by Two Separate Incisions

Due to the low Hg, 3 units of packed cells, 10 units Cryoglobulin and 3 units of FPP were transfused. On the 28th day after the surgery, the left side RSH was excluded as same as the right side. 3 units of PC and 10 units Cryoglobulin were transfused again. Furthermore, given the extent of hematoma and danger of infection, decision was made to perform surgery, and 2 separate incisions (Figure 2) were introduced, the extensive hematoma was evacuated, hemostasis was maintained. During the follow-up 6 months after the surgery the patient had no problem. 

## 4. Discussion

The occurrence of RSH has been estimated between 1.3-1.5 cases per year for the referrals to the radiology department (8). The ratio of the RSH in women is higher than men, numerally it is 2-3, it occurs in patients between ages 50-60 (9).

The most common presentations of RSH are acute abdominal pain and the appearance of a palpable mass. Should there be other symptoms such as nausea, vomiting, low grade fever, leukocytosis, severe hemorrhage or shock, the patient should consult with a physician (10).

In literature review diverse reasons are reported for the occurrence of the RSH. The prime cause is the use of anticoagulants, yet there are other factors such as abdominal trauma, previous surgery, asthma, blood pressure, pregnancy, intra-abdominal injection and after laparoscopy. Typically RSH occurs after severe coughs. The presented patient had undergone surgery and prior to the formation of RSH suffered from productive coughs.

The importance of RSH lies in the fact that it might be mistaken for different illnesses like appendicitis, bladder over-distention, splenomegaly, ovarian mass, sigmoid diverculitis, abrupt placenta, septic shock, myocardial infarction and cholecystitis (1). On the other hand, other diagnosis such as rupture in the aneurism of internal iliac artery should be considered for the early diagnosis of RSH (11). In the presented patient, given that the first transplant was performed on the right side, the early diagnosis was probable ruptured aneurism, but it was ruled out in later studies.

The diagnosis of RSH is conducted through sonography or CT scan but CT scan is the choice procedure (10). In our patient the CT scan rightly confirmed the diagnosis. RSH is usually unilateral; reports of a bilateral one are rare (4-6). As our knowledge, to date, no bilateral RSH has been reported in a kidney transplant patient.

Standard treatment of the limited RSH is pain control, bed rest along with tackling the problems associated (1). However, the successful arterial embolization has been cited in literature (12). Open surgery in the progressive hematoma, an uncertain diagnosis and in cases where patient is hemodynamically unsustainable and fluid resuscitation is ineffective, has indicated (10, 13).

With regard to the said patient, while hematoma was prominent in right side, we opted for medical treatment, but when it became bilateral, due to the extent of the hematoma and the danger of infection, we decided for open surgery.

Numerous side effects have been reported for RSH like hypovolemic shock, infection, the abdominal compartmental syndrome, myonecrosis, myocardial infarction, acute renal failure, ileus and death. Total mortality reported for RSH is %4 but in the iatrogenic cases it is %18 and in the cases induced by anticoagulants it even reaches %25. The mortality rates in pregnant women and the embryo are respectively %13 and %50 (10). Patient had no problem according to the follow-up six months after the operation.

## References

[1] Berna JD, Zuazu I, Madrigal M, Garcia-Medina V, Fernandez C, Guirado F (2000). Conservative treatment of large rectus sheath hematoma in patients undergoing anticoagulant therapy.. Abdom Imaging..

[2] Fitzgerald JE, Fitzgerald LA, Anderson FE, Acheson AG (2009). The changing nature of rectus sheath haematoma: case series and literature review.. Int J Surg..

[3] Cherry WB, Mueller PS (2006). Rectus sheath hematoma: review of 126 cases at a single institution.. Medicine (Baltimore)..

[4] Auten JD, Schofer JM, Banks SL, Rooney TB (2010). Exercise-induced bilateral rectus sheath hematomas presenting as acute abdominal pain with scrotal swelling and pressure: case report and review.. J Emerg Med..

[5] Docherty JG, Herrick AL (1991). Bilateral rectus sheath haematoma complicating alcoholic liver disease.. Br J Clin Pract..

[6] Yamada Y, Ogawa K, Shiomi E, Hayashi T (2010). Images in cardiovascular medicine. Bilateral rectus sheath hematoma developing during anticoagulant therapy.. Circulation..

[7] Franco A, Jimenez L, Munoz C, Chulia M, Marco P, Munoz E (2000). [Hematoma of the anterior rectus abdominis muscle as the first manifestation of lymphoproliferative disease after renal transplantation].. Nefrologia..

[8] Klingler PJ, Wetscher G, Glaser K, Tschmelitsch J, Schmid T, Hinder RA (1999). The use of ultrasound to differentiate rectus sheath hematoma from other acute abdominal disorders.. Surg Endosc..

[9] Osinbowale O, Bartholomew JR (2008). Rectus sheath hematoma.. Vasc Med..

[10] Gourgiotis S, Kotoulas D, Aloizos S, Kolovou A, Salemis NS, Kantounakis I (2009). Preoperative diagnosis of obscure gastrointestinal bleeding due to a GIST of the jejunum: a case report.. Cases J..

[11] de Donato G, Neri E, Baldi I, Setacci C (2004). Rupture of internal iliac artery aneurysm presenting as rectus sheath hematoma: case report.. J Vasc Surg..

[12] Rimola J, Perendreu J, Falco J, Fortuno JR, Massuet A, Branera J (2007). Percutaneous arterial embolization in the management of rectus sheath hematoma.. AJR Am J Roentgenol..

[13] Ruiz-Tovar J, Gamallo C (2008). Spontaneous rectus sheath hematoma: a case report.. Acta Chir Belg..

